# Do Synesthetes Have a General Advantage in Visual Search and Episodic Memory? A Case for Group Studies

**DOI:** 10.1371/journal.pone.0005037

**Published:** 2009-04-08

**Authors:** Nicolas Rothen, Beat Meier

**Affiliations:** Department of Psychology, University of Bern, Bern, Switzerland; Centre de Recherches su la Cognition Animale - Centre National de la Recherche Scientifique and Université Paul Sabatier, France

## Abstract

**Background:**

Some studies, most of them case-reports, suggest that synesthetes have an advantage in visual search and episodic memory tasks. The goal of this study was to examine this hypothesis in a group study.

**Methodology/Principal Findings:**

In the present study, we tested thirteen grapheme-color synesthetes and we compared their performance on a visual search task and a memory test to an age-, handedness-, education-, and gender-matched control group. The results showed no significant group differences (all relevant *p*s>.50). For the visual search task effect sizes indicated a small advantage for synesthetes (Cohen's *d* between .19 and .32). No such advantage was found for episodic memory (Cohen's *d*<.05).

**Conclusions/Significance:**

The results indicate that synesthesia per se does not seem to lead to a strong performance advantage. Rather, the superior performance of synesthetes observed in some case-report studies may be due to individual differences, to a selection bias or to a strategic use of synesthesia as a mnemonic. In order to establish universal effects of synesthesia on cognition single-case studies must be complemented by group studies.

## Introduction

In synesthesia the input of one modality activates brain areas which are normally not involved in processing inputs of that modality. This activation can result in an additional sensory experience, for example, a color experience for a black letter or a spoken word [1], [2]. Synesthesia is a phenomenon of great heterogeneity, in which a myriad of different stimuli (i.e., inducers) can trigger a myriad of different synesthetic experiences (i.e., concurrents). However, specific synesthetic associations are constant across time [3], [4]. One of the most studied forms is grapheme-color synesthesia in which graphemes trigger the experience of specific colors. Some case-report studies have found that grapheme-color synesthetes performed above average in visual search tasks and some single-case studies have also reported superior performance in episodic memory tests. The finding of a performance advantage in visual search suggests that synesthesia is a perceptual phenomenon. The reported superior memory suggests that synesthetes may have the opportunity to rely on additional retrieval cues compared to non-synesthetes. The goal of this study was to investigate in a group study whether grapheme-color synesthesia promotes a general performance benefit in visual search and memory performance. Before we present the new study, we give a brief overview of the relevant studies on visual search and on episodic memory.

### Visual Search

Ramachandran and Hubbard [5] investigated whether synesthetic experiences are genuinely perceptual rather than simple memory associations. Two synesthetes and forty control participants were tested with a visual search task. They were presented with displays that consisted of black graphemes, presented on a white background, for one second each. The displays were constructed such that some of the graphemes formed geometric shapes (i.e., a square, a rectangle, a triangle, or a diamond), which were embedded between other graphemes (i.e., distracters). The specific graphemes were selected such that for each synesthete the embedded shape and the distracter appeared either in red and green or in blue and yellow or vice versa. Participants had to indicate for each display which of the four figures was embedded. The results showed that synesthetes recognized more geometric shapes than the control group (81% vs. 59%), and Ramachandran and Hubbard [5] interpreted this result as evidence for the perceptual nature of synesthesia.

Palmeri [6] also conducted a visual search experiment in order to document the perceptual reality of synesthetic colors. They compared the performance of one single synesthete with a control group of seven non-synesthetes. The participants had to judge as quickly as possible whether a predefined target was present among a variable set of distracters (e.g., the digit 2 among several 5s). Displays consisted of 16, 25 or 36 digits which were presented in white on a black background in digital font. The control group showed a set size effect, that is, a linear increase of response times associated with increasing set size. The synesthete showed a significant smaller set size effect and responded significantly faster. Palmeri [6] concluded that synesthesia helps to promote visual search performance.

Laeng [7] conducted a similar study. Their main goal was to test whether the suggested advantage of synesthetes in visual search tasks is compatible with early-selection theories. One single synesthete and eight non-synesthetes had to search for a predefined target grapheme among predefined distracters of different set sizes. The results showed that if the targets had a similar form but elicited different synesthetic colors, controls but not the synesthete showed a significant set size effect. This finding supports the hypothesis that synesthetes may have a general advantage in visual search.

As far as we know, there are only three *group studies* in which synesthetes and controls were compared with visual search tasks. Hubbard [8] tested six synesthetes (one was already presented in the earlier study [5]). Five of them performed better than their respective controls in the visual search task. However, synesthetes performed significantly worse than controls who performed the task with colored displays. These results suggest that synesthetic colors improve visual search performance, but not as much as real colors for controls.

Edquist [9] compared 14 synesthetes with 14 matched control participants to examine whether synesthetic colors guide attention to the location of a target in an array of otherwise similar distracters. In a setup similar to the one used by Laeng [7] and Palmeri [6], the target graphemes were presented either in black or in a color that was congruent to the specific experience of individual synesthete. The results showed no performance advantage for synesthetes, neither for black nor for colored graphemes. Edquist [9] concluded that at least for the majority of individuals, synesthetic colors do not arise early enough in visual processing to guide or attract focal attention.

Gheri [10] compared seven synesthetes with seven controls in a condition where synesthetic colors were hypothesized to facilitate visual search performance and in a condition where the synesthetic colors were hypothesized to impair their performance compared to the control group. However, the results showed no performance differences between synesthetes and controls in any condition. Gheri concluded that colors arise rather at a cognitive than at a perceptual level. However, only the consistency of auditory grapheme color associations was tested for their participants. Therefore, the null-result regarding the visually presented graphemes in the visual search task must be treated with caution.

### Memory performance

Several single-case studies and some anecdotal observations indicate that synesthesia gives rise to an above average memory ability. For example, Cytowic [11] reported that synesthetes score in the superior range of the Wechsler Memory Scale. Many of them contended that their memory was excellent. Interestingly, they attributed the cause for their excellent memory to their synesthetic experiences, indicating for example, “I know it's two because it's white.” [11].

An example for a single-case with extraordinary memory was the famous mnemonist S. studied by Luria [12]. He was able to remember matrices of 50 digits after learning them for only a few minutes and he was able to remember these digits even years later. Besides the use of mnemonic techniques, Luria suggested that his extraordinary memory performance was at least in part caused by synesthesia [12] (but see [13] for a critical discussion).

Another single-case with exceptional abilities in numerical memory and mathematical calculations is the savant DT [14], [15]. Besides savantism DT has also an elaborate form of synesthesia for visually presented digits.

Moreover, Smilek [16] reported the case of synesthete C. who demonstrated an extraordinary capacity for remembering digits. C. and seven control participants were asked to learn three different matrices of 50 digits. The digits of one matrix were presented in black, those of another matrix were presented in colors that were congruent to C.'s synesthetic colors and those of the third matrix were presented in colors that were incongruent with C.'s synesthetic colors. C. showed an excellent memory performance for the black matrix and the one presented in congruent colors. However, C.'s recall for incongruently colored digits was very poor. For the control group there were no such differences in the recall of the three matrices. In addition, C. showed no decrease in recalling the matrix of black digits after 48 hours. In contrast, the performance of the control group was significantly poorer than their immediate recall.

Mills [17] tested memory performance of MLS, an individual with grapheme-color synesthesia and a matched control group. MLS reported that synesthesia helped her to remember names and other verbal material. Participants had to learn the names of 30 fictitious individuals (i.e., pairs of first and last names) for a paired-associates test. In addition, a number of standardized memory tests were administered: the Benton Visual Retention Test-Revised (BURT-R), the Rey-Osterrieth Complex Figure Test (CFT), and the Rey Auditory-Verbal Learning Test (RAVLT). The results showed that MLS scored higher in the verbal tests, that is, in the paired-associates test and in the RAVLT. However, her performance did not differ from the control group in the nonverbal memory tests (i.e., BURT-R and CFT). These results suggest that MLS was able to use her synesthesia to remember verbal materials, but they did not show a general memory performance benefit.

The only published group study in which the apparent memory benefit of grapheme-color synesthetes was investigated was conducted by Yaro and Ward [18]. They examined whether synesthetes reported higher memory ability than control participants and they also assessed what mnemonic techniques they used in a sample of 46 synesthetes and 46 non-synesthetes (Experiment 1). The results showed that synesthetes reported better memory than the control group. They also reported that they used visual strategies more often compared to the control group. In Experiment 2, several memory tests were administered to a subgroup of 16 synesthetes and a control group: the matrix test (congruent and incongruent colored digits), the RAVLT, the Farnsworth-Munsell color test, and the CFT. Compared to the control group, synesthetes showed better memory performance for colors and for words eliciting synesthetic colors and this advantage was more pronounced after a delay. However, there was no general advantage in the other tests. In addition, Yaro and Ward [18] did not replicate the memory advantage for congruent over incongruent stimuli reported by Smilek [16]. Overall the results showed that the performance benefit of synesthetes in memory tests is specifically related to color information.

### Interim Summary

In both domains, visual search and episodic memory, most of the studies were case-report studies and these studies demonstrate the superior performance of synesthetes. However, only very few studies have compared groups of synesthetes and these studies do not provide converging evidence for a general performance benefit. Case-report studies are important because they can demonstrate the existence of rare phenomena. However, they have the disadvantage that it is not easy to establish whether a statistical outlier was tested or whether there was some other selection bias (cf. [19]).

Testing a special case constricts the generalization of the results and therefore conclusions from single cases to a more general population have only limited value. The chance that a special case is tested is even more pronounced when inter-individual differences in the specific group of interest are large, and this is surely the case in synesthesia. Therefore, the evidence suggesting superior abilities in visual search and memory performance reported above may also reflect the heterogeneous constitution of grapheme-color synesthesia. For example, differences in the locus of color experience have lead to the distinction between associator and projector synesthetes. Associators report experiencing their photisms “in the mind's eye” whereas projectors report experiencing their photisms in external space [20]. Differences in the level of processing (i.e., conceptual vs. perceptual) have lead to the distinction between higher vs. lower synesthetes. In higher synesthetes it is the concept of graphemes that is critical for eliciting the synesthetic colors, in lower synesthetes it is the percept of graphemes that is critical for eliciting the synesthetic colors [21]. Importantly, however, none of these distinctions has been helpful in explaining why differences in cognitive performance do or do not occur in single case vs. group studies. Since Hubbard [8] did not collect phenomenological data concerning the projector-associator distinction this study is not informative on this issue. However, in the group study by Edquist [9] both projector and associator synesthetes were tested, and no performance advantage was found, neither overall nor for the particular projector synesthetes.

A selection bias may occur when an individual attracts the attention of a researcher by exceptional ability. Several single case studies have been conducted to scientifically demonstrate that some individuals are exceptional and the individuals have been included *because of their special performance in the first place*. S., tested by Luria [12], C., tested by Smilek [16], and MLS tested by Mills [17] all attracted the researcher's attention because of their extraordinary memory. It is obvious that this approach limits the generalization of the results. There is another type of selection bias reported by Hubbard and Ramachandran [22]. That is, synesthetes with particularly strong experiences may be more likely to approach researchers by themselves.

To summarize, it is not clear to date whether synesthesia causes a general advantage in visual search and episodic memory. To investigate this question, group studies are necessary. With increasing group size the probability that the results can be distorted by outliers or biased by selection decreases. To be clear, we do not want to fuel an old controversy on single-case vs. group studies (see [19], [23]), but we believe that group studies are necessary to solve the current question. Towards this goal we tested thirteen grapheme-color synesthetes, all of them associators, rather than a single case, and we compared their performance on the visual search task [5] and the matrix memory test [16], [18] to a yoked age-, handedness-, education-, and gender-matched control group. This sample was presented in an earlier study in which we demonstrated a synesthetic conditioning effect in the group of synesthetes but not in the control group [24]. Two testing sessions separated by two-to-three weeks were carried out in order to obtain a replication of the basic results for the visual search task and to test the trajectory of forgetting in the episodic memory test. The results revealed no significant performance advantage for synesthetes over the control group in either session, neither for visual search nor for episodic memory.

## Results

For the statistical analyses the significance level was set at alpha = .05. Cohen's d was used as a measure of effect size.

### Test of consistency

A computerized test of consistency [25], conducted in the original test session and in a retest session two-to-three weeks later, confirmed the participants' synesthesia. Participants had to choose a color for each grapheme from a color palette with 144 different colors. For the synesthetes, consistency was *r* = .94 for hue, *r* = .85 for saturation and *r* = .58 for value (brightness). For the controls, consistency was *r* = .21 for hue, *r* = .26 for saturation and *r* = .24 for value. All consistency estimates were higher for synesthetes than for controls, with *t*(24) = 7.83, *p*<.001 for hue, *t*(24) = 6.12, *p*<.001 for saturation, and *t*(24) = 2.32, *p*<.05 for value.

### Visual search task

In the visual search task participants were briefly presented with four different shapes (a square, a triangle, a rectangle or a hexagon) which were composed of graphemes that were embedded in a display of distracter graphemes. After each trial participants had to indicate which of the four forms was presented. Proportion of correct responses was analyzed.

**Figure 1:**
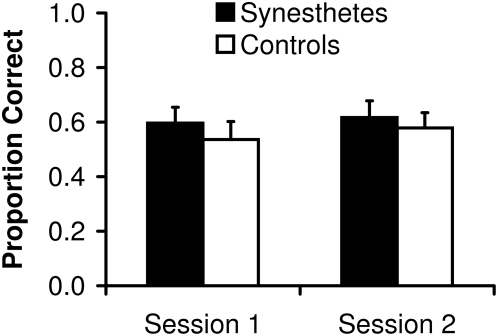
Visual search task: Performance of synesthetes and controls summarized for each session. Error bars represent standard errors.

The results are presented in Figure 1. Independent samples *t*-tests showed no significant group differences between synesthetes and controls, neither for the first session, *t*(24) = .68, *p* = .50, *d* = .27, nor for the second session, *t*(24) = .46, *p* = .65, *d* = .19.

**Figure 2:**
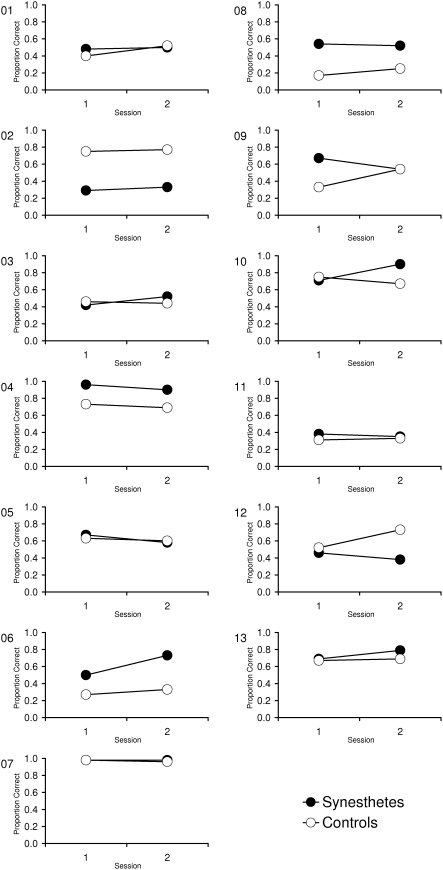
Visual search task: Performance of synesthetes and corresponding controls, for each individual separately.

One synesthete and the corresponding control participant performed close to ceiling (Figure 2; case 07). Therefore, these participants were excluded and an additional analysis was carried out. The mean proportion of correctly recognized shapes was .56 (*SD* = .18) for synesthetes and .50 (*SD* = .21) for controls in the first session and .59 (*SD* = .20) for synesthetes and .55 (*SD* = .17) for controls in the second session. Consistent with the prior analysis, *t*-test revealed no group differences neither for the first session, *t*(22) = .82, *p* = .42, *d* = .32, nor for the second session and *t*(24) = .52, *p* = .61, *d* = .23.

### Memory task

In the matrix memory test participants had to learn two different matrices consisting of 50 randomly generated graphemes for later recall. One consisted of black digits, the other consisted of digits that were incongruent to the concurrents of each individual synesthete. Proportion of correctly reproduced matrix cells was analyzed.

**Figure 3:**
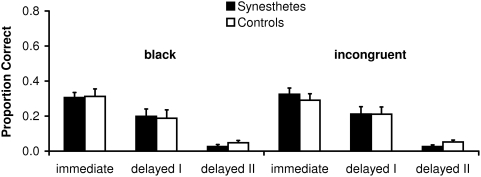
Memory task: Matrix Recall of synesthetes and controls summarized for each group. Error bars represent standard errors.

**Figure 4:**
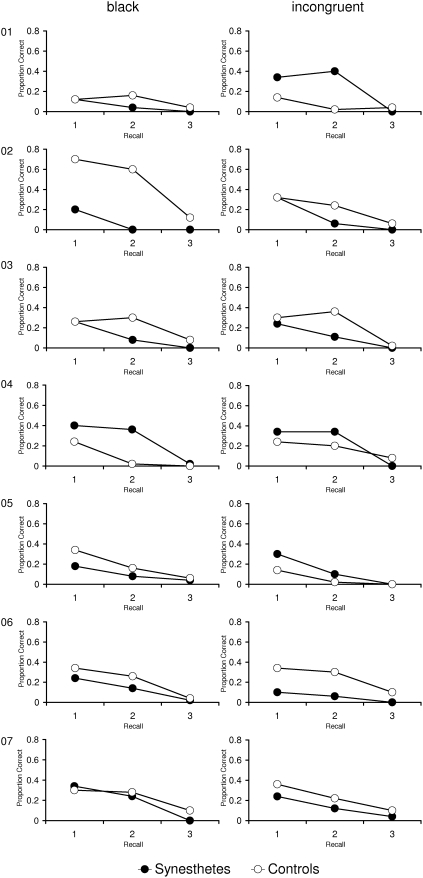
Memory task: Matrix Recall of synesthetes and corresponding controls, for each individual separately (Participants 1 to 7).

**Figure 5:**
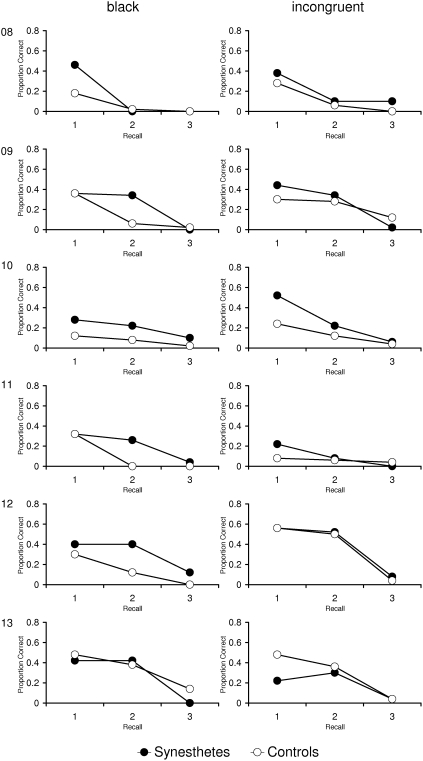
Memory task: Matrix Recall of synesthetes and corresponding controls, for each individual separately (Participants 8 to 13).

The results for each group are presented in Figure 3. Data of each individual synesthete and his/her yoked control person are presented in Figure 4 and Figure 5. A first inspection of the data revealed that there is no general advantage in episodic memory for synesthetes compared to controls. A mixed three-factorial analysis of variance (ANOVA) with Group (synesthetes, controls) as between-subjects factor and Recall Phase (Immediate Matrix Recall, Delayed Matrix Recall I, Delayed Matrix Recall II) and Grapheme Color (black, incongruent) as within-subject factors revealed a significant main effect of Recall Phase, *F*(2,98) = 96.4, *p*<.01. Most importantly, neither the main effect of Group, *F*(1,24)<.01, *p* = .97, *d* = .01, nor any interaction involving Group approached significance (Group×Recall Phase, *F*(2,48) = .51, *p* = .60; Group×Grapheme Color, *F*(1,24) = .04, *p* = .84; Group×Recall Phase×Grapheme Color, *F*(2,48) = .45, *p* = .64).

We reanalyzed the data for the first two Recall Phases (Immediate Matrix Recall, Delayed Matrix Recall I) because performance after the two to three week interval was at floor (Delayed Matrix Recall II). The mixed three-factorial analysis of variance (ANOVA) with Group (synesthetes, controls) as between-subjects factor and Recall Phase (Immediate Matrix Recall, Delayed Matrix Recall I) and Grapheme Color (black, incongruent) as within-subject factors revealed a significant main effect for Recall Phase (*F*(1,24) = 36.67, *p*<.01. Again and most importantly, neither the main effect of Group, *F*(1,24) = .05, *p* = .83; *d* = .05, nor any interaction involving group approached significance (Group×Recall Phase, *F*(1,24) = .05, *p* = .82; Group×Grapheme Color, *F*(1,24) = .07, *p* = .80; Group×Recall Phase×Grapheme Color, *F*(1,24) = .98, *p* = .33).

## Discussion

The goal of this study was to investigate whether synesthesia promotes cognitive abilities such as visual search and episodic memory. Towards this goal we tested a group of 13 grapheme-color synesthetes. A test of consistency confirmed the true nature of their synesthesia. For each synesthete a control person was selected that was matched for age, gender, handedness and education. Visual search and memory performance were assessed with tasks that have been previously used in synesthesia research. The results showed no general performance benefit for synesthetes, neither for visual search nor for episodic memory.

For the visual search task, the group means showed a tendency towards a performance benefit for the group of synesthetes although the participants were associators. A closer inspection of the individual data revealed that this tendency was essentially caused by three individuals who outperformed their yoked controls (Figure 2; synesthete 04, 06 and 08). Would we have tested only these three synesthetes (or even only one of them), we would clearly have come to the conclusion that synesthesia *does* promote performance in visual search. In addition, our results combined with other findings from the literature might indicate that *some* synesthetes have an advantage in visual search. Specifically, two studies that found a performance advantage in synesthetes for visual search tasks were conducted with projectors [5], [6]. In contrast our study was conducted with associators. Accordingly, one could conclude that the probability that synesthetes show the pop out effect in visual search tasks is higher for projectors than for associators. However, the data reveal also that one individual of the control group outperformed her respective synesthete (Figure 2; control 02). Therefore, an alternative interpretation is that the results may be caused by individual differences which are independent of synesthesia. The results also indicate that the presence of a performance advantage in visual search cannot be used as a diagnostic for true synesthesia. Neuroimaging methods and physiological measures like the synesthetic conditioning test seem to be more appropriate towards this goal (cf. [1], [24], [26], [27]).

For the episodic memory test, there was no evidence for a performance benefit for synesthetes in the present study, neither at the level of group means nor at the individual level. Performance in the delayed recall was rather low and the synesthetic conditioning task which was administered during the retention interval may have contributed to this result. However, the test procedure was identical for synesthetes and controls, therefore, no differential effects can have emerged for synesthetes and controls.

Moreover, we did not find an influence of the matrix type (black vs. incongruent). This result contrasts the findings from the single-case study by Smilek [16]. They showed that synesthete C. performed lower when the material consisted of digits that were incongruently colored to her synesthetic colors. However, the present study replicates a finding of Yaro and Ward [18] who also did not find a difference in memory performance for congruently and incongruently colored digit matrices. It is possible that C. is a synesthete who experiences very strong photisms, which can cause behavioral effects not found in other synesthetes. However, Smilek [16] reported only the scores of the first attempt (out of four) for the recall of the incongruent matrix. Therefore, it is possible that C. was able to compensate strategically on the other trials.

To summarize, the conclusions regarding a general performance benefit for synesthetes on episodic memory are similar to those regarding visual search. Most studies that found better memory performance in synesthetes were single-case studies. In the cases of S., C. and MLS it is clear that they were selected on the basis of their extraordinary abilities concerning memory performance a priori [12], [16], [17]. It is important to note that the authors of these studies do not claim that synesthesia per se leads to extraordinary memory performance. Nevertheless these studies suggest that synesthesia promotes this extraordinary ability. The fact that other studies did not find clear evidence for extraordinary memory in synesthetes may reflect inter-individual differences between different synesthetes. These differences may influence how synesthetes use their synesthesia. It is very likely that a deliberate use of synesthesia as a mnemotechnique is critical for the expression of extraordinary memory in synesthetes.

Overall, our results indicate that on the group level a performance benefit in visual search and memory performance can fail to appear even with genuinely true synesthetes. This suggests that the superior performance of synesthetes in single-case studies may be rather due to strategic use of synesthesia and general individual differences than due to synesthesia per se. Another possibility is that different sub-types of grapheme-color synesthesia exist and that the classification must be refined in order to do justice to the many variants of synesthesia (cf. [8], [20]). It is possible that a combination of the associator vs. projector, higher vs. lower distinction with other dimensions, for example the degree of intentional use of synesthesia in every-day life may provide a helpful framework.

To date, it is likely that a publication bias exists, because studies that find differences between synesthetes and controls are probably more likely to be published than studies that do not find such differences. However, to enhance our understanding of the impact of synesthesia on cognition and to establish general performance benefits case-report studies should be complemented by group studies.

## Materials and Methods

### Participants

We tested 13 grapheme-color synesthetes (7 female and 6 male, *M* = 24.15 years, *SD* = 4.14) and 13 controls (7 female and 6 male, *M* = 23.62 years, *SD* = 4.11). Controls were matched for age, gender, handedness and education. The synesthetes were classified as associators because they all reported to experience the colors before their mind's eye according to a questionnaire published by Ward and Simner [28]. A computerized test of consistency [25] was conducted in the original test session and in a retest session two-to-three weeks later to confirm the participants' synaesthesia. Ten synesthetes had color experiences for all letters and digits (N = 36), one synesthete had color experiences for letters only (N = 26), one synesthete had color experiences for digits only (N = 10) and one synesthete had color experiences for digits and two letters (N = 12). Participants took part in this study voluntarily, they were fully informed about the purpose of this study, and they were informed that they can withdraw and terminate their participation at any time during the study. All participants provided verbal consent. No IRB approval was required for this type of psychological research.

### Apparatus and Materials

#### Visual search task

This task was modeled according to Ramachandran and Hubbard [5]. Stimulus material consisted of four different shapes (a square, a triangle, a rectangle or a hexagon) composed of graphemes embedded in a display of two other graphemes. Graphemes were presented in black on a white background. They were chosen individually for each synesthete such that he/she experienced the shape and the two distracter graphemes either as red in green, green in red, yellow in blue or blue in yellow (see Table S1). Each display consisted of 44 to 48 graphemes. The task consisted of a total of 48 trials. Two different sets of target-distractor combinations were used in each session. Stimuli were presented with E-Prime 1.1 software [29] on an IBM-compatible computer with a 15-inch VGA monitor.

#### Memory task

This task was modeled according to Smilek [16]. It consisted of two matrices, each separately printed on a white paper sheet. Each matrix included 50 randomly generated digits (0 to 9) printed in 10 rows and 5 columns. The same digit was never placed as an immediate neighbor. Digits were 0.3 cm wide and 0.6 cm high. One matrix consisted of black digits. The other matrix consisted of digits that were incongruent with the concurrents of each individual synesthete. For each synesthete and his/her yoked control participant, the digits of the incongruent matrix were printed in the same colors. For all participants the colored matrix consisted of the same digits. For synesthetes, the colors of the digits were adjusted individually for the incongruent matrix. One synesthete experienced colors only for letters. Thus, her matrices and the matrices of her corresponding control were composed of letters. Letter matrices were generated analogous to the digit matrices. For the recall tests a 10×5 matrix printed on white paper was used.

### Procedure

Participants were tested in two sessions, which were separated by an interval of two-to-three weeks. Table 1 shows the ordering of activities for both sessions.

**Table 1:** Ordering of activities. The interval between test sessions was two-to-three weeks.

Session 1	
1.1	Test of Consistency
1.2	Visual Search
1.3	Matrix Study Phase
1.4	Immediate Matrix Recall
1.5	Retention Interval
1.6	Delayed Matrix Recall I
**Session 2**	
2.1	Test of Consistency
2.2	Visual Search
2.3	Delayed Matrix Recall II

#### Visual search task

Participants were seated 60 cm in front of the computer screen. They were instructed to search for embedded figures. Specifically, they were told that the embedded figure may be a square, a rectangle, a triangle, or a hexagon. First, participants performed three practice trials. Each trial consisted of a sequence of five screens. First, the instruction “space = start” was displayed until the participant pressed the spacebar to initiate the trial. Then the word “attention” was displayed for 1500 ms and changed to “attention!” for another 500 ms. Then the stimulus display was presented for 1000 ms. Then the stimulus disappeared and a screen appeared on which the participant indicated which shape he/she recognized by pressing the appropriate key. If they did not recognize any shape, they were instructed to guess by pressing one of the four keys to get to the next trial.

#### Memory task

In the first test session, the Matrix Study Phase, the Immediate Matrix Recall and the Delayed Matrix Recall I were administered. Each participant was presented with a matrix of black graphemes for three minutes with the instruction to memorize the graphemes and their positions. In the Immediate Matrix Recall participants had to recall all the graphemes of the black matrix. Then the Matrix Study Phase and the Immediate Matrix Recall for the incongruent matrix was administered analogously. The interval between the Matrix Study Phase/Immediate Matrix Recall and the Delayed Matrix Recall I was filled with the synesthetic conditioning task which lasts about 30 min. The procedure and the results of this task have been presented elsewhere [24]. Then the Delayed Matrix Recall I was administered. First, participants were asked to recall the matrix of black graphemes and then they had to recall the colored matrix. The second test session consisted of three parts: The consistency test, the visual search task and the Delayed Matrix Recall II.

#### Synesthetic conditioning task

In this task participants are presented with colored displays across three different phases (i.e., habituation, conditioning, and extinction). In the conditioning phase one specific color is followed immediately by a loud startling sound which served as the unconditioned stimulus. The critical comparison involves trials on which the sound was not present: trials with the conditioned color only and trials on which the letter of the trained color-letter association is presented. In a previous study we have demonstrated that synesthetes, but not controls, showed a conditioned response to graphemes that elicited the conditioned synesthetic color [24].

## Supporting Information

Table S1 Individual stimulus sets for the visual search task. Each subject was presented with both sets in each session.
(0.04 MB DOC)
