# Supplementary material for: Do Synesthetes Have a General Advantage in Visual Search and Episodic Memory? A Case for Group Studies
Source: PLoS One. 2009 Apr 8;4(4):e5037. doi: 10.1371/journal.pone.0005037 (PMC2660420; doi:10.1371/journal.pone.0005037)
Supplement: Table S1 — Individual stimulus sets for the visual search task. Each subject was presented with both sets in each session. (0.04 MB DOC) [file pone.0005037.s001.doc]

## Supplementary Table

*Table S1.* Individual stimulus sets for the visual search task. Each subject was presented with both sets in each session.

| **Subject** | **Set 1** | | **Set 2** | |
| --- | --- | --- | --- | --- |
|  | **Target** | **Distracters** | **Target** | **Distracters** |
| 01 | a | b, d | l | i, j |
| 02 | h | b, d | o | c, e |
| 03 | 6 | 3, 5 | a | b, e |
| 04 | c | a, e | A | V, Y |
| 05 | 7 | 5, 9 | E | P, R |
| 06 | 5 | 2, 3 | C | D, F |
| 07 | 7 | 2, 8 | 7 | 3, 6 |
| 08 | 3 | 5, 6 | e | a, p |
| 09 | 8 | 2, 6 | 9 | 5, 6 |
| 10 | 2 | 4, 8 | 4 | 3, 5 |
| 11 | 5 | 3, 9 | B | D, S |
| 12 | 3 | 2, 5 | 8 | 6, 9 |
| 13 | A | N, X | B | C, E |
